# Short- and potential long-term adverse health outcomes of COVID-19: a rapid review

**DOI:** 10.1080/22221751.2020.1825914

**Published:** 2020-10-07

**Authors:** T. Y. M. Leung, A. Y. L. Chan, E. W. Chan, V. K. Y. Chan, C. S. L. Chui, B. J. Cowling, L. Gao, M. Q. Ge, I. F. N. Hung, M. S. M. Ip, P. Ip, K. K. Lau, C. S. Lau, L. K. W. Lau, W. K. Leung, X. Li, H. Luo, K. K. C. Man, V. W. S. Ng, C. W. Siu, E. Y. F. Wan, Y. K. Wing, C. S. M. Wong, K. H. T. Wong, I. C. K. Wong

**Affiliations:** a Centre for Safe Medication Practice and Research, Department of Pharmacology and Pharmacy, LKS Faculty of Medicine, University of Hong Kong, Hong Kong Special Administrative Region, People’s Republic of China; b Department of Paediatrics and Adolescent Medicine, LKS Faculty of Medicine, University of Hong Kong, Hong Kong Special Administrative Region, People’s Republic of China; c Department of Social Work and Social Administration, Faculty of Social Science, University of Hong Kong, Hong Kong Special Administrative Region, People’s Republic of China; d WHO Collaborating Centre for Infectious Disease Epidemiology and Control, School of Public Health, LKS Faculty of Medicine, University of Hong Kong, Hong Kong Special Administrative Region, People’s Republic of China; e Department of Medicine, LKS Faculty of Medicine, University of Hong Kong, Hong Kong Special Administrative Region, People’s Republic of China; f Department of Computer Science, Faculty of Engineering, University of Hong Kong, Hong Kong Special Administrative Region, People’s Republic of China; g Research Department of Practice and Policy, UCL School of Pharmacy, London, UK; h Department of Family Medicine and Primary Care, LKS Faculty of Medicine, University of Hong Kong, Hong Kong Special Administrative Region, People’s Republic of China; i Department of Psychiatry, Faculty of Medicine, Chinese University of Hong Kong, Hong Kong Special Administrative Region, People’s Republic of China; j Department of Medicine, Queen Mary Hospital, Hong Kong Special Administrative Region, People’s Republic of China

**Keywords:** COVID-19, SARS-CoV-2, coronavirus infection, health outcomes, complications, short-term adverse outcomes, long term adverse outcomes

## Abstract

The coronavirus disease 2019 (COVID-19) pandemic has resulted in millions of patients infected worldwide and indirectly affecting even more individuals through disruption of daily living. Long-term adverse outcomes have been reported with similar diseases from other coronaviruses, namely Middle East Respiratory Syndrome (MERS) and Severe Acute Respiratory Syndrome (SARS). Emerging evidence suggests that COVID-19 adversely affects different systems in the human body. This review summarizes the current evidence on the short-term adverse health outcomes and assesses the risk of potential long-term adverse outcomes of COVID-19. Major adverse outcomes were found to affect different body systems: immune system (including but not limited to Guillain-Barré syndrome and paediatric inflammatory multisystem syndrome), respiratory system (lung fibrosis and pulmonary thromboembolism), cardiovascular system (cardiomyopathy and coagulopathy), neurological system (sensory dysfunction and stroke), as well as cutaneous and gastrointestinal manifestations, impaired hepatic and renal function. Mental health in patients with COVID-19 was also found to be adversely affected. The burden of caring for COVID-19 survivors is likely to be huge. Therefore, it is important for policy makers to develop comprehensive strategies in providing resources and capacity in the healthcare system. Future epidemiological studies are needed to further investigate the long-term impact on COVID-19 survivors.

## GRAPHICAL ABSTRACT

**Figure:**
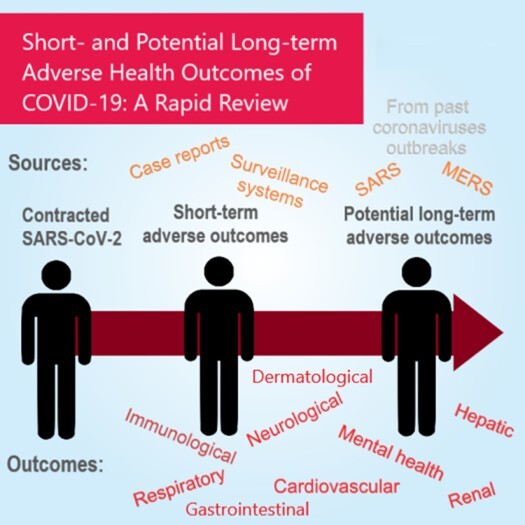

## Introduction

Coronavirus disease 2019 (COVID-19) is caused by severe acute respiratory syndrome coronavirus 2 (SAR-CoV-2) infection. As of 28 July 2020, 16,341,920 COVID-19 cases and 650,805 deaths worldwide have been reported [1]. While the global community is focusing on trialling COVID-19 vaccines and acute treatment, it is equally important to evaluate the long-term consequence of COVID-19 survivors. By 28 July 2020, 10,451,291 people had recovered from COVID-19 [2]. Despite the increasing number of recovered cases, there is rising concern regarding the sequelae following a diagnosis of COVID-19 [3]. Emerging evidence indicates COVID-19 has long-term consequences on the immunological and respiratory systems. Furthermore, since SARS-CoV-2 is a recently discovered novel virus, there is a lack of information on the long-term health outcomes of COVID-19 survivors. As the pathogens responsible for recent coronaviruses outbreaks, namely severe acute respiratory syndrome (SARS) and Middle East respiratory syndrome (MERS), are similar in genome structures and are closely-related phylogenetically [4], long-term health outcomes of SARS and MERS may shed light on the long-term consequences of COVID-19. In this review, we summarize current evidence on the short-term adverse health outcomes and assess the risk of potential long-term adverse effects of COVID-19 referencing to long-term adverse outcomes of SARS and MERS. We will also recommend future research areas for long-term health outcomes of COVID-19 survivors.

## Search strategy, selection criteria and narrative review

References were identified by conducting literature search using PubMed up till June 2020. Additional references were identified from the citations of relevant articles. Keywords used include COVID-19, SARS-CoV-2, SARS-CoV, MERS-CoV. Details of the keywords for each organ system are included in the supplementary material. The keywords were used in combination with MeSH terms to extend the comprehensiveness of the literature search. Only human studies were reviewed. No language restrictions were applied in the search. The references were included based on relevance to the topics of interest.

We divided the narrative review into different sections according to organ class systems. The evidence of each section was reviewed by an expert clinician and two biomedical scientists. A narrative review of each section was written by the trios. The first author and corresponding author were responsible for summarizing all the information into the final manuscript.

## Narrative review

### Immunological outcomes

SARS-CoV-2 is believed to induce much of its pathology through immune-mediated processes. Immunological consequences were extensively reported during the acute phase of illness, including lymphopenia, reduced peripheral blood T cells and increased plasma proinflammatory cytokines [5]. Historically, rheumatological and autoimmune-related adverse effects were manifested in patients following previous outbreaks, related but not limited to Zika, Ebola, Chikungunya and influenza viruses [6]. The most widely discussed theoretical explanation for such association between viral infection and the adverse immunological outcomes is suggested by molecular mimicry, the cross-talking of epitope between the host and the virus which favours the activation of autoreactive humoral or cell-mediated immunity. Other potential mechanisms to disrupt self-tolerance include bystander activation and damage, epitope spreading and the ineffective clearance of persistent viruses [6,7]. SARS-CoV-2 may induce its pathology through a similar mechanism, however, further studies are needed to support such hypothesis.

#### Guillain-Barré syndrome and paediatric inflammatory multisystem syndrome

Guillain-Barré syndrome (GBS), where the hyperactive immune system attacks neurons as a para-infectious or post-infectious phenomenon, mainly manifesting as ascending muscle weakness, has been reported in over 30 case reports. Details are presented in Appendix. This phenomenon is consistent with preclinical studies that showed coronaviruses had the potential to induce demyelinating diseases [8]. To the best of our knowledge, no study has investigated the causal relationship between COVID-19 and GBS, and it is unknown if the neurological consequences of GBS could worsen the prognosis of the former. In children, despite their usual milder clinical course, cases of paediatric inflammatory multisystem syndrome (PIMS) which is a collection of Kawasaki-like disease, toxic shock syndrome and myocarditis, have been described [9]. More evidence is required to delineate whether COVID-19 occurred coincidentally or consequentially with autoimmune diseases and their natural history. There is an increasing number of case reports on GBS and PIMS among COVID-19 cases, with 52 cases and over 700 cases reported respectively. The published case reports are presented in the Appendix. However, as information on PIMS cases are too heterogeneous to generate informative summary statistics, while only case reports were published on GBS, future epidemiological studies are needed to investigate their incidences and monitor for any persistent adverse outcomes.

#### Haematological complications

In addition to GBS and PIMS, case reports of other autoimmune and autoinflammatory diseases also suggest that there might be associations between COVID-19 and adverse immunological outcomes, including immune thrombocytopenic purpura (ITP) and antiphospholipid syndrome, affecting haemostasis as well as increasing the risk of thrombosis [10].

Two published studies detailed the rates of venous thromboembolism in patients with COVID-19. The incidence of venous thromboembolism in patients with severe COVID-19 or requiring intensive care was reported as 25% in a study in China [11], and 31% in another study from the Netherlands [12]. Features of disseminated intravascular coagulation (DIC) and pulmonary embolism were also highly prevalent in COVID-19, where DIC has been observed in 71.4% of non-survivors [13]. The phenotype of pulmonary embolism in COVID-19 is observed to be different from conventional thromboembolism, and postulated to represent in situ immunothrombosis [14]. Whether pulmonary thromboembolism in COVID-19 resolves completely in survivors or poses long term sequelae of lung parenchymal or pulmonary vascular damage or pulmonary hypertension remains unknown. The haematological complications in patients with COVID-19 are also reflected in the cerebrovascular events observed, details are reported in the neurological section. As different ethnicities have different thrombotic risk [15], international studies are needed to investigate the varying risks of adverse outcomes in different ethnicities. Epidemiological studies with longer follow-up time are also needed to assess the long-term outcomes.

#### Other autoimmune diseases and immunological consequences

A cohort study using a national database of 17 million patients in the UK reported an increased risk of mortality (adjusted hazard ratio, 1.23, [95% CI, 1.12-1.35]) in hospitalized COVID-19 patients with autoimmune diseases such as rheumatoid arthritis, systemic lupus erythematosus, and psoriasis [16]. Another review study hypothesized that COVID-19 infection may increase the risk of cancer in COVID-19 survivors due to impaired immune responses [17]. It is important to continue monitoring the occurrence of immunological and autoimmune diseases and evaluate the risk of cancer among COVID-19 survivors, which may take years to develop. Given the suggested linkage of COVID-19 and induced immunological diseases, long term epidemiological studies to identify new post-infectious immunological sequelae are required.

### Respiratory outcomes

The lungs remain the most common organ of serious injury as short-term outcomes of COVID-19, manifesting as pneumonia and respiratory failure which is the major cause of mortality in the acute phase [18]. Asymptomatic patients with COVID-19 may have evidence of pneumonia on CT imaging [19]. Patients who survive are at risk of chronic respiratory complications. According to the British Thoracic Society Guidance on respiratory follow up of patients with a clinical diagnosis of COVID-19 pneumonia, those with severe conditions have a high prevalence of post-viral lung fibrosis, pulmonary thromboembolism, and attendant functional impairment [20].

Available studies have consistently demonstrated that residual abnormalities on chest CT scans are present in many COVID-19 survivors discharged from hospital 4–6 weeks after the onset of illness [19]. In a study of COVID-19 survivors in Italy, while 72.7% of the subjects developed interstitial pneumonia during hospitalization, up to 43.4% reported residual dyspnoea at around one month post-discharge, the second most prevalent symptom after fatigue (53.1%) [21], even though these latter symptoms may be multi-factorial and not solely due to lung damage. Early reports indicate that impairment of lung function test parameters, mainly the carbon monoxide diffusing capacity, is seen at 4–6 weeks from the onset of illness in those who have recovered and discharged from hospital [22]. Details about pulmonary embolism and its persistence in COVID-19 survivors are reported in the section for immunological outcomes.

#### Potential long-term adverse respiratory outcomes

From experience of previous outbreaks of SARS and MERS, radiologic abnormalities, impairment of pulmonary functions and reduced exercise capacity improved over time but may be persistent in some for months or even years [23,24]. Follow-up studies of SARS patients showed long-term persistent radiographic abnormalities in the lungs in a minority [24], while 33% of MERS survivors showed radiographic evidence of pulmonary fibrosis at 82.4 ± 66 (mean ± SD) days after discharge [23]. It is reasonable to hypothesize that some COVID-19 patients may suffer from adverse respiratory outcomes despite recovery. Studies are needed to monitor the course of residual lung damage such as lung fibrosis, bronchiectasis or other structural abnormalities shown on radiologic imaging, and to estimate the impact of COVID-19 infection on subsequent lung health, pulmonary function, exercise capacity and health-related quality of life among survivors. Factors that determine persistent respiratory impairment in survivors are worthy of exploration, in particular the use of anti-viral agents, systemic corticosteroids, other immunomodulating or anti-inflammatory agents, advanced life support with invasive or non-invasive ventilation or extracorporeal membrane oxygenation.

### Cardiovascular outcomes

The high expression of angiotensin-converting enzyme 2 (ACE2) receptors on myocardial tissue renders cardiomyocytes highly susceptible to attack by SARS-CoV-2 [25]. Indeed, myocardial injury is an often reported cardiovascular complication of COVID-19, with an average incidence of 8-12% [26]. According to the National Health Commission of China, almost 12% of patients without known cardiovascular diseases (CVD) demonstrated an elevation in troponin levels or developed cardiac arrest during hospitalization [27].

In a Wuhan cohort of 150 patients with COVID-19, myocardial damage and heart failure accounted for 40% of deaths [28]. In another cohort of 191 patients, heart failure occurred in 52% and 12% of patients who subsequently died and were discharged respectively [29]. Cardiac arrhythmia is another common cardiovascular manifestation of COVID-19. A study reported an overall incidence of 16.7% in 138 Chinese patients with COVID-19, with higher incidence of arrhythmia (44.4%) in those admitted to the ICU than those not requiring ICU admission (8.9%) [30]. In a cohort of 21 severe COVID-19 cases, one-third developed cardiomyopathy [31]. DIC was also observed in non-survivors of COVID-19, details are reported in the section for immunological outcomes.

#### Potential long-term adverse cardiovascular outcomes

Even after recovery, heightened systemic inflammatory and pro-coagulant activity can persist long after resolution of the index infection which may lead to adverse cardiovascular outcomes in the long-term. Previous meta-analysis and studies with long-term follow-up have shown that patients with community-acquired pneumonia are at significantly greater risk of cardiovascular and cerebrovascular diseases in the long run, even if they do not have underlying CVD at baseline [32]. Indeed, amongst 25 SARS survivors, 40% continued to have cardiovascular abnormalities at the 12-year follow-up [33]. It is likely that patients with COVID-19 related pneumonia will experience similar adverse outcomes. Interestingly, cardiovascular abnormalities observed during hospitalization in eight patients with H7N9 influenza returned to normal at 1-year follow-up [34]. Future longitudinal follow-up of COVID-19 survivors would be crucial to elucidate its long-term impact of COVID-19 and to protect these patients from future CVD.

### Gastrointestinal, hepatic and renal outcomes

Gastrointestinal (GI) symptoms are often found in COVID-19 patients. The pooled prevalence of GI symptoms from 29 studies was found to be 15% [35], with up to 26% reported in some studies [36]. The most prevalent symptoms were diarrhoea, nausea and vomiting, and abdominal pain [35]. ACE2 receptors are also abundantly expressed on intestinal enterocytes, making them susceptible to attack by SARS-CoV-2 [36]. A study found that COVID-19 patients with GI symptoms had a similar rate of complications, pharmacological agents and supportive care received, hospitalizations and mortality as those without GI symptoms, indicating that GI symptoms are not a strong prognostic factor for outcomes in COVID-19 [37]. However, SARS-CoV-2 RNA were found in faecal specimens of 54% of COVID patients [35], and were continuously detected in 23.29% of the patients after negative results were seen in respiratory samples [38]. This suggests that SARS-CoV-2 may persist longer in enterocytes than in respiratory epithelial cells. Future epidemiological studies are needed to monitor for long-term adverse GI outcomes in COVID-19 patients.

Published case studies have reported COVID-19 patients with varying degrees of liver dysfunction [35]. Liver injury primarily presented with aberrant elevation in alanine aminotransferase, aspartate aminotransferase and bilirubin levels [35]. The severity of COVID-19 has been suggested to be positively correlated with liver dysfunction in available data, where liver damage in mild cases of COVID-19 appears to be transient [35], with elevation in liver enzymes normalizing upon discharge or subsequent follow-up [39]. Liver involvement was also reported in SARS [40] and MERS [41] patients. However, no reports on their long-term outcomes have been found. With the paucity of data, it remains unclear whether such liver injury observed in COVID-19 as well as SARS and MERS infection were inflammation-mediated or from direct viral infection. A recent multi-centre North American matched cohort study showed that cirrhotic patients with COVID-19 had similar mortality to cirrhotic patients alone, and Charlson Comorbidity Index was the only independent predictor for mortality [42].

Similarly, kidney involvement is also prevalent in COVID-19 patients. Incidence of acute kidney injury (AKI) was around 0.5%–29% in patients with COVID-19 [43]. Along with proteinuria and haematuria, AKI was a risk factor for severity and mortality of COVID-19 [43]. In COVID-19 patients from Europe and the US who required intensive care, AKI was found in 20%–40% of these cases [44]. However, the actual incidence of AKI remains uncertain, as most studies did not report AKI stages nor had a clear definition of AKI. A clinical study is underway to investigate the incidence of renal outcomes in patients with COVID-19 three months post-hospital admission [45]. Kidney involvement was also found in SARS [46] and MERS [47] patients. However, no studies on the long-term renal outcomes have been reported. AKI significantly contributes to long-term outcomes such as microalbuminuria, and chronic kidney diseases that require dialysis [48]. As such, these outcomes should be monitored alongside investigation on the role of early initiation of renal replacement and sequential extracorporeal therapies in patients with COVID-19.

### Neurological outcomes

Many studies have been published on the neurological impact of COVID-19. Olfactory and/or gustatory dysfunction are the most common neurological manifestations reported in patients with COVID-19 [49], with the pooled prevalence of 52.73% for olfactory dysfunction and 43.93% for gustatory dysfunction reported from a recent meta-analysis [50]. It is proposed that SARS-CoV-2 binds to ACE2 receptor, which triggers a series of signalling cascades and potentially leads to different adverse neurological outcomes, e.g. olfactory dysfunction after entering the brain by systemic circulation or through the nasal cavity [51].

There are also case reports on the occurrence of rare events such as stroke [52], GBS [53], and encephalitis/encephalopathy [54]. 57 cases of ischemic stroke and 9 cases of intracerebral haemorrhage were notified within the first three weeks of the launch of a nationwide surveillance system on neurological complications of COVID-19 in the United Kingdom (UK) [55]. Stroke was also found to be a poor prognostic factor in patients with COVID-19. A systematic review found that COVID-19 patients with stroke as a complication had a three times higher risk of death [56]. Another meta-analysis [57] also found that the severity of COVID-19 was 2.5-fold higher in patients with a history of cerebrovascular disease. Details on GBS are reported in the above section for immunological outcomes. However, there is limited information on whether patients experiencing such symptoms have a higher risk of other severe neurological outcomes [8]. Population-based studies with longer follow-up time are needed to assess the impact of COVID-19 on rare but severe outcomes.

For those neurological outcomes that can produce long-term complications, such as stroke and encephalitis which may seriously affect the patient's later quality of life [58], more studies on the risk factors of such complications, in the form of population-based studies with larger sample size and longer follow-up time are needed.

### Dermatological outcomes

Various cases of diverse dermatological manifestations of the COVID-19 have also been reported. The incidence of the cutaneous manifestation in COVID-19 patients varies in different case series, possibly due to the under-recognition of those asymptomatic or pauci-symptomatic cases, ranges from 0.2% to 20.4% depending on regions of origin [59]. Proposed mechanisms of how SARS-CoV-2 affect dermatological system include direct viral attack on the epidermal basal cells and vasculature endothelial cells, with ACE2 expression in skin keratinocytes as possible target, and indirectly by the inflammatory response to the virus [60].

A report of 88 COVID-19 patients in Italy showed 20.4% of them developed cutaneous manifestations, mostly involving erythematous rash, seen over a wide spectrum of clinical appearances including macular, papular, maculopapular, erythema-multiforme- like eruptions, [59]. Nearly half of the patients developed cutaneous symptoms at onset of disease. A nationwide surveillance system reported 375 cases of cutaneous lesions within two weeks during early April in Spain [61]. Maculopapular lesions were the most common presentations, making up nearly half of the cases. Pseudo-chilblain and urticarial lesions were the next most common presentations, each making up 19%, while vesicular lesions (9%) and livedo or necrosis (6%) making up the rest [61]. Cases of oral ulcers and blistering [62] and herpetiform lesions [63] were also reported. Dermatological manifestations were also reported in children. Rash is also a common manifestation in children with PIMS, mimicking Kawasaki-pattern. Details on PIMS are reported in the above section on immunological outcomes.

Onset of adverse dermatological outcomes spanned across the course of COVID-19, ranging from presenting as the prodromal symptoms to a few days after systemic symptoms onset, or in the cases of PIMS, to delayed manifestations a few weeks after recovery for the infection [59,61,64]. A case-series of 22 patients who developed varicella-like exanthem during the course of COVID-19 reported onset from two days before to 12 days after systemic symptoms [64]. The duration of cutaneous symptoms were generally short and resolved within a few days [65]. Asymptomatic carriers would jeopardise the epidemiological control of disease in the communities. Recognition of the cutaneous symptoms as the first and sole manifestations of COVID-19 in some individuals [66] may help in early detection of disease, especially in areas when diagnostic kits are limited.

For the projection of long-term outcomes based on SARS and MERS, no reports of dermatological outcomes in both diseases were found. As dermatological examinations are often not routinely conducted in patients with coronaviruses, the prevalence of adverse dermatological outcomes in COVID-19 patients may be under-reported [61,62]. Larger epidemiological studies are needed to assess the impact of COVID-19 on rare but severe dermatological outcomes.

### Mental health outcomes

On top of the immediate and long-term damage to the physical health brought by COVID-19 to patients, the COVID-19 pandemic is having a profound effect on the mental health of COVID-19 survivors across the globe [67]. The virus may infect the brain or trigger an immune response that causes additional adverse effects on brain functions and mental health [68]. Mental disorders might be the sequelae of brain damage that are either triggered directly from cerebral hypoxia caused by the viral infection [69], or indirectly from the immunological response or immunotherapy-induced adverse effects [70].

#### Potential long-term adverse mental health outcomes

As observed from previous large-scale coronavirus pandemics like SARS and MERS, post-traumatic stress disorder (PTSD), depression and anxiety disorder were much prevalent in patients with SARS or MERS at the post-illness stage [69]. The most common symptoms during the acute phase of illness among patients who were hospitalized for SARS or MERS were insomnia (41.9%), followed by impaired concentration or attention (38.2%), anxiety (35.7%), impaired memory (34.1%) and depressed mood (32.6%) [69].

Similar findings were observed in studies conducted in different regions or countries at different timepoints during or post-pandemic [71–73]. In Hong Kong, compared to the baseline of 3% having psychiatric morbidities, a marked increase in the prevalence of mental disorders at 42.5% was found among SARS survivors at around 4-year post-SARS period, including PTSD (25.6%), depressive disorders (16.6%), anxiety disorder (15.5%), somatoform pain disorder (15.5%), panic disorder (13.8%) [72]. Chronic fatigue, associated with more comorbid active psychiatric disorders, was also found in 40.3% SARS survivors [72]. In South Korea, 54% of MERS survivors were reported to have at least one symptom of PTSD, depression, suicidality, or insomnia [73]. However, there were some common limitations among these studies, including small sample sizes, short follow-up time, and the outcome measurements were based on self-reported questionnaires or surveys instead of diagnosis given by the clinicians. Thus, the findings might not be a comprehensive reflection of the true phenomenon. High-quality international research is needed to systematically examine the mental health effects of the pandemic on COVID-19 patients in a longer term [67].

### Potential adverse maternal outcomes and long-term outcomes of prenatal exposure to COVID-19

Previous reports in Hong Kong have portrayed associations of SARS with critical maternal illness such as multi-organ failure and disseminated intravascular coagulation, miscarriage in early pregnancy, or maternal death [74]. In addition, studies have reported stillbirth and worsened prognosis in pregnant women infected with MERS [75]. A case report showed that two infants from five pregnant women with SARS presented growth retardation and two manifested GI complications [76]. A population-based cohort study conducted in the UK at the early stage of the pandemic reported that 5% of infants from mothers tested positive for SARS-CoV-2 RNA, potentially contracted from their infected mothers [77]. While the current limited evidence does not seem to point to overtly poor immediate outcomes to children born to mothers with COVID-19, it is unclear whether there are any potential long-term effects [77]. Converging evidence from both clinical and preclinical research indicates that changes in the maternal gestational immune environment can alter foetal brain development and increase the risk for certain neurodevelopmental disorders [78]. As COVID-19 is a novel infectious disease which affects the immune system significantly in some patients, using an international big-data network will provide us with a large sample size to monitor and explore the potential long-term effects of COVID-19 on children’s central nervous system, such as autism spectrum disorder and attention deficit hyperactivity disorder. Therefore, an international surveillance system must be developed to monitor the long-term outcomes of prenatal COVID-19 exposure.

## Discussion

Our review has found that COVID-19 has been reported with various adverse outcomes in different systems in the body. A lot of the adverse health effects were mediated by the activated immune system in response to the virus. GBS and PIMs were two of the immune-related syndromes that were more frequently reported. The activated immune system has also led to haematological complications in various forms, including ITP, antiphospholipid syndrome, affecting haemostasis or thrombosis, which in turn manifests in vascular diseases including pulmonary thromboembolism, haemorrhagic and ischaemic stroke. Another concern arises from the activated immune system is the increase in risk of long-term adverse immunological outcomes, including autoimmune diseases and cancer. Adverse respiratory outcomes can be immune-mediated or due to the direct attack of the virus, including lung fibrosis and functional impairment, which has been found to persist beyond recovery from COVID-19 in some patients. Based on experience with SARS and MERS, impaired pulmonary function can persist years after recovery from infection, leading to reduced exercise capacity and quality of life. In the acute phase of the infection, we found reports of adverse cardiovascular outcomes including myocardial damage, heart failure, arrhythmia and adverse neurological outcome including olfactory and gustatory dysfunction. Based on experience with other pneumonia, cardiovascular abnormalities and elevated risk of cardiovascular and cerebrovascular events persists beyond a decade after the infection. For adverse dermatological, gastrointestinal, hepatic and renal outcomes, only complications during the acute phase of the infection were reported. No reports of their long-term outcomes in SARS and MERS survivors were found. Adverse mental health outcomes were also reported during the pandemics, including insomnia, anxiety, and depressed mood. Increase in risk of some adverse mental health outcomes could persist years after recovery from infection, including chronic fatigue, PTSD, depression, anxiety, panic disorder etc. The adverse outcomes we found and their potential long-term outcomes from direct exposure and prenatal exposures calls for comprehensive and long-term care and monitoring.

Post-infection care for COVID-19 survivors is likely to add an extra burden to the healthcare system. It was reported that healthcare utilization of a thousand SARS survivors in Hong Kong during the first year after hospital discharge was substantial, with over 5,000 attendances for consultations in primary care clinics and diagnostic tests, including chest radiographs and blood tests [79]. Some patients required inpatient rehabilitation or underwent surgical procedures over the course of the follow-up year [79]. Assuming a similar impact of COVID-19 on healthcare utilization, the estimated additional numbers of clinic visits and diagnostic tests will be over 45 million among the current 9 million recovered COVID-19 patients worldwide (to July 2020). Given increasing numbers of recovered COVID-19 patients and aging compared to the time of the SARS outbreak, it is likely that the extent to which follow-up healthcare services are adopted for COVID-19 survivors will be much greater than for SARS globally. Prolonged use of mechanical ventilation, defined as a duration of more than 96 h to 21 days of ventilation or longer, is associated with higher mortality rates and increased healthcare utilization [80]. Given the extensive use of mechanical ventilation in severe COVID-19 patients, post-infection healthcare utilization is projected to be substantial in these survivors. Therefore, it is important for policy makers to develop a comprehensive strategy in providing resources and capacity in caring for COVID-19 survivors.

The vast majority of COVID-19 survivors have less than six months of follow-up after recovery; hence there is no data on long-term outcomes of COVID-19 survivors or the children with pre-natal exposure of SARS-CoV-2. Taking the above knowledge gaps into consideration, it is clear that methodologically, clinical trials will not be sufficient to resolve all questions on the long-term physical health and mental health outcomes of patients with COVID-19 and children with prenatal exposure to SARS-CoV-2. Therefore, the application of epidemiological and statistical techniques using large clinical and administrative records databases is the most appropriate option to evaluate the long-term health outcomes of COVID-19. Big data analytics will enable evaluation of the effects of COVID-19 to better understand the outcomes of patients who have recovered from the acute phase of COVID-19. This will enable us to gain a more comprehensive understanding of the immediate impact of COVID-19 on individual patients. Such analytics could also identify the risk factors of poor outcomes, which will enable the development of strategies to protect patients who are most vulnerable to severe illness. Initiatives such as International COVID-19 Data Research Alliance and Workbench and OHDSI – Observational Health Data Sciences and Informatics will significantly enhance the capacity of research in long-term outcomes of COVID-19 and also, more importantly, avoid unnecessary duplications of effort and wasted precious resources. Such initiatives could provide empirical evidence from healthcare big data to introduce evidence-based clinical practice for patients post-COVID-19 care. The information extracted would give educators and policymakers a high contrast full picture illustration of the impact of a pandemic on the holistic development of COVID-19 survivors.

Most of the available literature are case reports, data from surveillance systems or studies with limited cohort size or relatively short study period or follow-up time. The study design and the small number of cases in each report gives insufficient power to allow for proper quantitative analysis to draw statistically meaningful and generalisable conclusion. This review referenced some meta-analyses of other reports to provide an overview of a specific adverse outcome. However, due to the sheer volume and variety of papers and data available, it was not possible to use checklists to assess the accuracy or quality of each article whether they are meta-analyses or case reports. This is a major limitation of our review; inevitably all narrative reviews subject to the same limitation. Nevertheless, in order to enhance the validity of our narrative review, each section was written by subject specialist. Such strategy aims to make use of the specialist knowledge to select more appropriate information under the current limitation.

## Conclusion

This review of current evidence on the short-term and potential long-term health outcomes of COVID-19 showed that multiple organ systems as well as mental health are affected by the COVID-19 pandemic. The burden of caring for COVID-19 survivors are likely to be huge. Therefore, it is important for policy makers to develop a comprehensive strategy in providing resources and capacity in the healthcare system. Future epidemiological studies are needed to further investigate the risk factors associated with adverse outcomes in patients with COVID-19 and monitor the long-term health impact.

## Supplementary Material

COVID19.adverse.outcomes.review_v5.7.3.5_4_clean.docx

COVID_adverse_outcomes_review_Appendix_200911.docx
