# Supplementary material for: Short- and potential long-term adverse health outcomes of COVID-19: a rapid review
Source: Emerg Microbes Infect. 2020 Oct 7;9(1):2190–9. doi: 10.1080/22221751.2020.1825914 (PMC7586446; doi:10.1080/22221751.2020.1825914)
Supplement: COVID_adverse_outcomes_review_Appendix_200911.docx [file TEMI_A_1825914_SM1645.docx]

Appendix 1

Search terms

| **Topic** | **Keywords** |
| --- | --- |
| COVID-19 | severe acute respiratory syndrome coronavirus 2, 2019-nCoV, SARS-CoV-2, COVID-19 |
| Previous coronaviruses | long-term outcome of SARS, long-term outcome of MERS |
| Immunological | immunology, Guillain Barre Syndrome, interstitial lung disease, arthritis, anti-phospholipid antibody syndrome, autoimmune disease, multisystem inflammatory syndrome in children |
| Respiratory | respiratory, lung, pulmonary |
| Cardiovascular | cardiovascular diseases, coronary disease, coronary artery disease, heart failure, myocardial infarction, heart attack, myocardial ischemia |
| Neurological | encephalitis, myelitis, olfactory disorder, ageusia, cerebrovascular event, stroke, neurology |
| Gastrointestinal | gastrointestinal, stomach, intestine, inflammatory bowel disease |
| Hepatic | hepatic, liver |
| Renal | kidney diseases, renal replacement therapy, nephrology, proteinuria |
| Dermatological | dermatology, cutaneous, skin |
| Mental health | mental health, mental disorder, psychiatric disorder, mental status, psychological, neuropsychiatric |
| COVID-19 = coronavirus disease 2019 | |
